# An enormous Italian pedigree of Marfan syndrome with a novel mutation in the FBN1 gene

**DOI:** 10.1002/ccr3.2881

**Published:** 2020-06-02

**Authors:** Omid Daneshjoo, Leila B. Salehi, Antonio Pizzuti, Giuseppe Novelli, Federica Sangiuolo

**Affiliations:** ^1^ Medical Genetics Group Department of Experimental Medicine “Sapienza’’ University of Rome Rome Italy; ^2^ U.O.C. of Medical Genetics Policlinic of Tor Vergata Rome Italy; ^3^ Rare Diseases Centre for Marfan Syndrome and Related Disorders Policlinico Tor Vergata Rome Italy; ^4^ Department of Biomedicine and Prevention University of Rome “Tor Vergata’’ Italy

**Keywords:** *FBN1*, Marfan syndrome, mutation, NGS

## Abstract

We characterize a large Italian family presenting with Marfan syndrome (MFS), where the same NM_000138.4:c.6872‐1G > T splice site mutation in the FBN1 gene was detected in 37 affected individuals with different pathological phenotypes. Further studies on such a large pedigree could identify other genetic factors that influence MFS manifestation.

## Graphical abstract

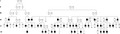

## 1 INTRODUCTION

Marfan syndrome (MFS) is a hereditary connective tissue disorder and causes mainly by a mutation in the *FBN1* gene. In this research, *FBN1* was screened for all affected individuals in a suspected Italian pedigree to the MFS.

Marfan syndrome (MFS) is a hereditary connective tissue disorder with skeletal, cardiovascular, and ocular manifestations. The inheritance pattern in this syndrome is autosomal dominant (AD), and mutations in the gene for fibrillin‐1 (FBN1) cause most cases of MFS. This gene is responsible for the production of fibrillin‐1 glycoprotein in the human body.^1^, ^2^, ^3^, ^4^, ^5^

Incidence of MFS is approximately 1 in 5000 births, without differences among gender, ethnic, and geographic groups of affected individuals. In 75% of cases, this syndrome has a hereditary cause (25% results of new mutation).^6^, ^7^

The location of *FBN1* in the human genome is on the long arm of chromosome 15 (15q21.1) including 66 exons and approximately 200 kbp size.^5^, ^8^ Most of the reported causative mutations in MFS‐affected patients have been missense mutation with a dominant‐negative effect which results in <35% of the expected production of fibrillin‐1 protein in the extracellular matrix.^9^ Splice site mutations and deletions in *FBN1* are the other kinds of mutations in MFS with a lower proportion.^10^ At least 25% of Marfan syndrome cases are the result of a new mutation in *FBN1*.^3^, ^11^

The extensive variable clinical phenotypes have seen in this syndrome in both intrafamiliar and unrelated patients, even with the same mutation in *FBN1*.^6^

Tall stature, long limbs, anterior chest deformity, scoliosis, high arched palate, and joint laxity are the widespread phenotype manifestations in this syndrome. Lens subluxation of the eye is an important clinical ocular finding in the affected individuals.^12^ The most common pulmonary involvement in MFS is spontaneous pneumothorax.^13^ Interstitial fibrosis in the apices is a rare phenomenon among affected individuals which has been described as an extreme example of MFS manifestation. In contrast, restriction on the pulmonary system has commonly seen in MFS patients which causes by skeletal abnormalities such as pectus deformities and scoliosis.^14^

Evaluation of cardiovascular complications has an important value due to the direct involvement of the mentioned difficulties in the affected individuals’ survival. The cardiovascular complications could express in severe forms including aortic aneurysm, aortic ectasia, mitral and aortic valvular regurgitation with acute aortic dissection.^15^ Dilatation of the ascending aorta in the proportion of MFS‐affected patients is susceptible to lead to aorta dissection which causes life‐threatening situations.^16^

The correlation between genotype and phenotype in MFS is weak in many cases, and there are numerous examples of patients with a different manifestation of complication in skeletal, cardiovascular, ocular, pulmonary, and connective tissues, even in the affected families with the same type of mutations.^9^, ^17^, ^18^

Detailed diagnostic criteria, referred to as the revised Ghent criteria, are in general use by geneticists. In the modern era, clinical criteria which were published in 1986 (Berlin) brought up to date in 1996 and revised in 2010.^10^, ^19^

## 2 CASE PRESENTATION

**Figure 1:**
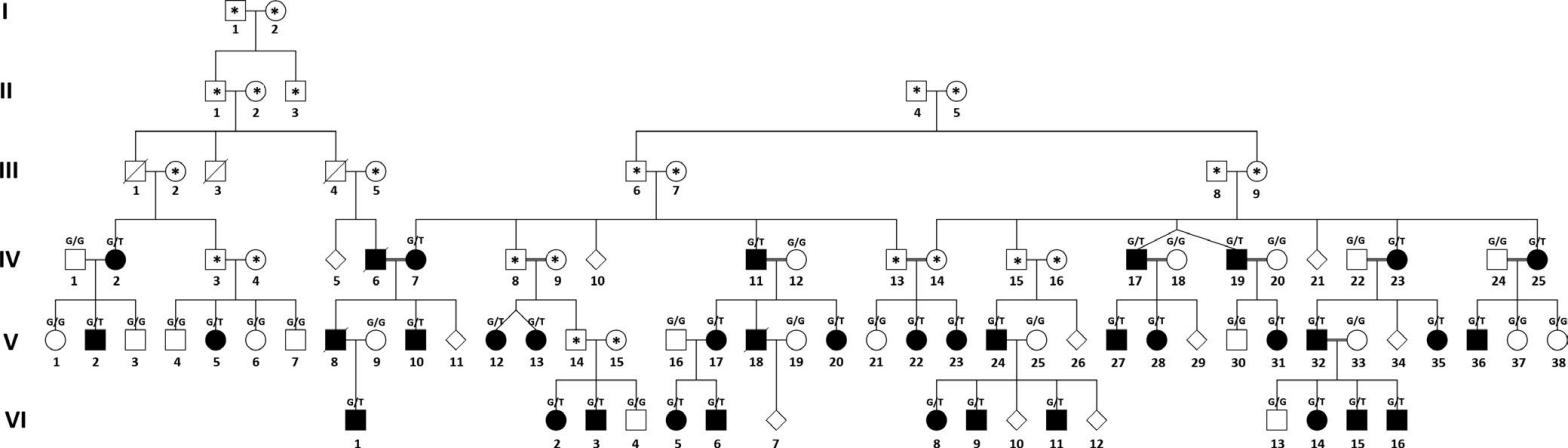
The pedigree of studied family for Marfan syndrome (*means due to inaccessibility, evaluation of marked individuals were not possible, G/G = Homozygous Normal Phenotype, G/T = Heterozygous Abnormal Phenotype)

The individuals in one MFS‐suspected Italian pedigree were investigated for the mutation screening in the *FBN1*. Specifically, 37 affected individuals to MFS were referred to the multidisciplinary Marfan clinic in Tor Vergata University Hospital, Rome, Italy (“SIMaRaLCode” 12092003), from 2008 to 2017 (Figure 1).

All participants, or their legal guardians, provided written and informed consent. The institutional review boards of the Sapienza University of Rome and the University of Rome “Tor Vergata’’ were reviewed the project, and all experiments were performed following relevant named guidelines and regulations.

The mentioned patients were evaluated by cardiologists/cardio‐surgeons, ophthalmologists, orthopedics, and odontoid‐stomatologists at the beginning. All children with the age of ≤14 years (n = 15) were referred to pediatricians as well. Cardiac ultrasonography and slit‐lamp examinations were systematically performed for each subject. Skeletal involvement was evaluated by the X‐ray examination after the orthopedic evaluation for each subject. After performing all the mentioned special examinations, patients were taken a calculated total MFS score and then were taken the genetic counseling before performing the genetic test. The blood samples and the informed consent for genetic tests were obtained from all patients during the final session. All 37 patients were fulfilled for the criteria of the Revised Ghent Nosology.

The genomic DNA of all patients isolated from 200 µL whole blood by using EZ1 DNA Blood Kit and an EZ1 Advanced XL automatic extractor (QIAGEN GmbH) following the manufacturer's instructions. The DNA concentration was estimated by using Qubit^®^ dsDNA HS Assay Kit (Thermo Fisher) and a Qubit^®^ 2.0 Fluorometer.

A custom genetic panel including coding regions and exon‐intron boundaries of the *FBN1* was designed by using Ion AmpliSeq™ (Designer software: Thermo Fisher). Two different pools of primers were obtained for all amplicons. The calculated coverage of the coding sequence with a minimum coverage depth of 20× was 97%, with exon padding of 15 bp. The libraries were generated by 20 ng of DNA by using Ion AmpliSeq Library Kit v2.0 and following the manufacturer's instruction. The generated libraries were indexed by using the Ion Xpress Barcode Adapter Kit. After dilution of the samples at 100 pmol/L, libraries were pooled for emulsion PCR by Ion OneTouch™‐2 machine using Ion PGM™ Template OT2 200kit according to the manufacturer's instruction. Ion Sphere™ particles were enriched by the Ion OneTouch™ enrichment System. The amplified templates were sequenced by using an Ion Torrent PGM platform and 318v2 chip. All the sequencing instruments and reagents were supplied by Thermo Fisher, Foster City, CA.

The binary Alignment/Map (BAM) and variant call format (VCF) files were generated by using the plugin preinstalled Torrent browser. The BAM files were analyzed by using IGV (Integrative Genomics Viewer, www.broadinstitute.org/igv) and Ion Reporter™ software (Thermo Fisher).

The in silico pathogenicity prediction of the rare variants was performed by using the following software: MutationTaster (http://www.mutationtaster.org/) and Human Splicing Finder (http://www.umd.be/HSF3/). The mutation nomenclature was established according to the HGVS recommendations, version 15.11. The “NM_000138.4” reference sequence was used for the genetic screening in this research.

The allele frequency of the identified causative mutation in *FBN1* was determined to show the rare frequency of the mentioned variant in healthy public population by using the following public databases: dbSNP Common 144 (NCBI), 1K Genome project phase 3 (www.1000genomes.org), Exome Aggregation Consortium version 0.3 (ExAC), and UK Biobank.

The genetic conservation of the region harboring the identified mutation was surveyed by comparing that region of the genome in Human, Dog, Rhesus, Mouse, Elephant, Chicken, and several other vertebrates in UCSC and ConSurf databases.^20^, ^21^, ^22^

An ABI 3130xl‐automated sequencer (Applied Biosystems) was used either to confirm the obtained variant that identified by NGS method and for sequencing the regions which were not covered by the NGS. The NM_000138.4:c.6872‐1G > T variant also was sequenced for the familial segregation analysis by using the Sanger sequencing method. The previously published primers were used for sequencing the regions of interest in *FBN1*.^23^ The Sequencing chromatograms were analyzed by using CodonCode Aligner software.

**Figure 2:**
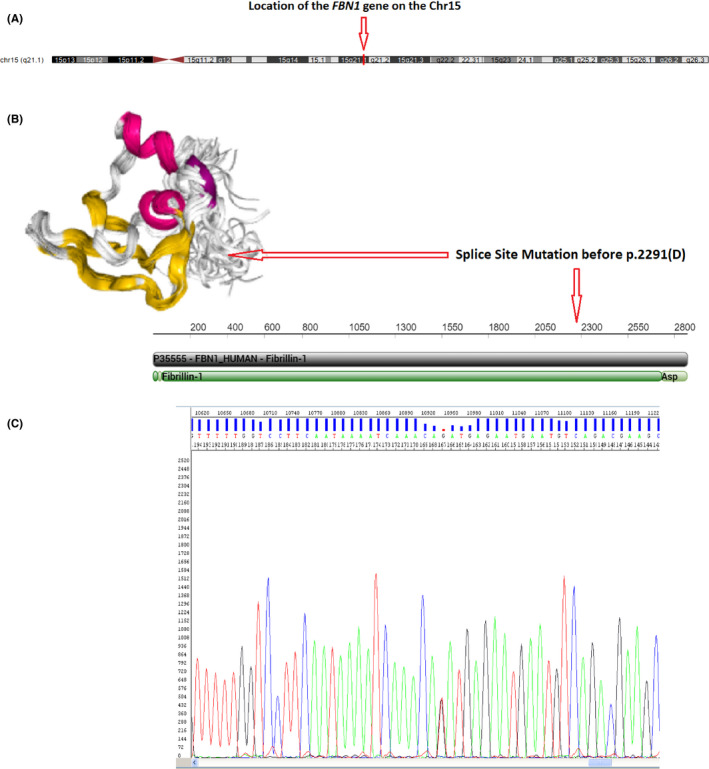
Schematic depiction of *FBN1* Localization on Chr15 (A), position of identified mutation on the *FBN1* protein production, and its location in the mentioned protein amino acid sequence (B). Sanger sequencing electropherogram of the proband (C)

The revealed causative mutation was a novel NM_000138.4:c.6872‐1G > T splice site mutation (Figure 2), Chr15: 48720669C > A (GRCh37.p13). The mentioned mutation which was revealed by the NGS method was an identical genetic alteration among all the affected individuals and approved by the ClinVar database accession criteria (ClinVar submission ID: SUB6800706). The NGS result was confirmed by the Sanger sequencing method successively, among all affected individuals in the studied pedigree.

The exact location of the revealed mutation in the *FBN1* gene and location of the probable disturbance in the structure of protein product and its aa sequence (if the mutant gene has protein product) are depicted in a schematic form in Figure 2.

The clinical condition of patients and phenotypes’ detail is described in Table 1 which shows the fulfillment of Revised Ghent nosology for all affected individuals.

**Table 1:** Clinical signs of patients for Revised Ghent nosology: (+) indicates fulfillment and (−) indicates nonfulfillment

Pedigree position	Age	Z‐score, kind of heart surgery (2018)	Ectopia Lentis	Systemic score ≥ 7	*FBN1* mutation	The satisfaction of the Revised Ghent nosology
IV‐2	47	0.79	−	+	+	+
IV‐6	47	Bentall operation	−	+	+	+
IV‐7	47	David operation	−	+	+	+
IV‐11	56	3.2	−	+	+	+
IV‐17	43	David	−	+	+	+
IV‐19	43	David	−	+	+	+
IV‐23	62	3.41	−	−	+	+
IV‐25	60	2.08	−	+	+	+
V‐2	26	5.99	−	+	+	+
V‐5	13	2.65	−	+	+	+
V‐8	25	Bentall operation	−	+	+	+
V‐10	21	1.68	−	+	+	+
V‐12	16	0.74	−	+	+	+
V‐13	16	0.43	−	+	+	+
V‐17	32	2.2	−	−	+	+
V‐18	39	4.53	−	−	+	+
V‐20	46	0.55	−	+	+	+
V‐22	27	1.41	−	+	+	+
V‐23	28	2.68	−	+	+	+
V‐24	32	David operation	+	+	+	+
V‐27	9	2.85	−	+	+	+
V‐28	22	0.4	−	−	+	+
V‐31	12	0	−	+	+	+
V‐32	38	David operation	−	+	+	+
V‐35	22	3	−	−	+	+
V‐36	38	4.5	−	+	+	+
VI‐1	8	6.44	−	+	+	+
VI‐2	6	2.18	−	+	+	+
VI‐3	7	3.36	−	−	+	+
VI‐5	9	0.42	+	−	+	+
VI‐6	12	0.12	−	+	+	+
VI‐8	6	1.83	−	+	+	+
VI‐9	10	1.2	−	+	+	+
VI‐11	11	1.36	+	+	+	+
VI‐14	14	0.8	−	+	+	+
VI‐15	14	0	−	+	+	+
VI‐16	18	1.18	−	+	+	+

Note

The systemic score indicates systemic involvement (score ≥ 7) (+) and no systemic involvement (score < 7) (−). Z‐score for cardiac involvement which indicates aortic root enlargement, Z‐score ≥ 2.0 for subjects age 20 y and above, and Z‐score ≥ 3.0 for subjects younger than age 20 y indicates fulfillment for Cardiac involvement. Ocular involvement includes ectopia lentis (EL).

**Figure 3:**
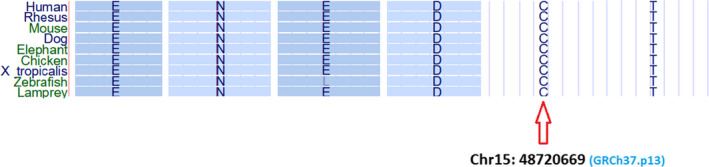
Schematic depiction of mentioned locus conservation among Human, Rhesus, Mouse, Dog, Elephant, Chicken, X tropicalis, Zebrafish, and lamprey

Multiple alignments for the region of this mutation in the several species such as Human, Dog, Rhesus, Mouse, Elephant, and Chicken showed that the location of this mutation is highly conserved, arguing that this bp plays an important role in the production of the mRNA and the protein product (Figure 3).

The in silico pathogenicity prediction tools indicated that the revealed novel variant is a pathogen variant, furthermore the identified novel NM_000138.4:c.6872‐1G > T *FBN1* variant is classified as a pathogen variant according to the segregation analysis, ACMG (American College of Medical Genetics and Genomics) standard guidelines and the Revised Ghent nosology.^10^, ^24^

### 2.1 Result of this assay

Statistical analysis of patient clinical information and mutation screening results was undertaken in an attempt to elucidate genotype‐phenotype correlations.

## 3 DISCUSSION

One of the unexplained features of MFS is the pathogenic mechanism which leads to the variable phenotype manifestation despite the complete penetrance in MFS. The variable phenotype expression and recently extended different diagnostic criteria, confounded making the accurate diagnostic decision for MFS‐suspected patients. In this study, there were several MFS‐affected individuals with variable phenotypes and distinctive systemic complications, even in the one family. For example, patient IV‐2 had a mutation in the *FBN1* and did not show any cardiovascular sign on the evaluation time at age 47, while cardiovascular involvement was seen at age 26 for his offspring during the clinical evaluation (Figure 1). These observations and general difficulties in definition of MFS in affected patients, due to overlapping of this syndrome with several related conditions like the MASS and MVPS, suggest the *FBN1* mutation screening in the suspected individuals as an effective method for complete MFS diagnosis, which is also clearly advised in the revised Ghent nosology.^10^

It is reported over 3000 different mutations in the *FBN1* have been revealed until this time. These mutations classify as two main types including dominant‐negative type (DN) and haploinsufficiency type (HI). Generally, DN mutations in this gene cause folding disturbance of the protein product due to the composition of the mutant gene product with the normal fibrillin‐1 product. Alternatively, the HI mutations cause loss of the production of the mutant gene.^6^

It has been reported MFS patients with a dominant‐negative mutation in *FBN1* are more susceptible to express ectopia lentis which argues that the evaluation of this sign is beneficial for early diagnosis of this syndrome.^9^, ^25^ Although several cohort studies have tried to make a connection between genotype and phenotype in MFS‐affected individuals, the establishment of this correlation has remained unclear until now.

In this research, which was performed in one of the largest MFS pedigrees in Italy with a high grade of consanguinity, the observation of mentioned occasion cardiovascular manifestation in affected members of the family provokes suspicion of the involvement of separate genetic elements in MFS manifestation in addition to *FBN1* mutations. In one recent research, the importance of particular genetic factors involving the mutations in cardiovascular‐related genes has been evaluated as an effective element in addition to the mutations in the *FBN1*. The result showed that these mutations were involved in alteration of the phenotype manifestation in the MFS‐affected patients.^26^ The result of this research suggests the further investigation of this MFS pedigree to find the other factors which are probably involved in the mentioned cardiovascular phenotype manifestation. The more severe condition of MFS phenotypes in the offspring of inbred mating is the other suggestive evidence for the fact that mutations in the related genes in addition to *FBN1* mutations can affect the manifestation of the MFS phenotype.

## 4 CONCLUSION

The studied MFS‐affected pedigree in this research is one of the largest Italian MFS pedigrees with 37 affected members and interesting history of recurrent inbreeding. The c.6872‐1G > T mutation in the *FBN1* was the cause of MFS in the affected members of this pedigree which indicates that *FBN1* mutation screening in the suspected individuals is an important part of MFS diagnosis procedure, according to the revised Ghent nosology. Observation of the occasion cardiovascular manifestations in the affected members of the family and the fact that the affected individuals shared an identical *FBN1* mutation are the suggestive evidence for additional prospective researches to reveal additional involved elements in variable phenotype manifestation of MFS.

## CONFLICT OF INTEREST

In this study, the authors have no conflict of interest.

## AUTHOR CONTRIBUTIONS

OM: analyzed the data, wrote, and reviewed this report. LB. S and FC. S: collected the data. GN and AP: contributed to the interpretation of data. All authors: approved the final version of the manuscript.

## ETHICAL APPROVAL

All procedures were under the ethical standards of the national research committee.

## Data Availability

The result of genetic analysis is available in the ClinVar database (ClinVar submission ID: SUB6800706).
